# From silicon to silica: a green chemistry approach for hollow sphere nanoparticle formation†

**DOI:** 10.1039/d4na00586d

**Published:** 2024-10-11

**Authors:** Hennie Marie Johnsen, Anuj Pokle, Werner Filtvedt, Marianne Hiorth, Jo Klaveness, Anja Olafsen Sjåstad

**Affiliations:** a Department of Pharmacy, University of Oslo Sem Sælands vei 3 0371 Oslo Norway h.m.johnsen@farmasi.uio.no marianne.hiorth@farmasi.uio.no jo.klaveness@farmasi.uio.no; b Nacamed AS Oslo Science Park, Gaustadalléen 21 0349 Oslo Norway wf@nacamed.com; c Department of Physics, Center for Materials Science and Nanotechnology, University of Oslo POB 1048 Blindern 0316 Oslo Norway anuj.pokle@fys.uio.no; d Department of Chemistry, University of Oslo Sem Sælands vei 26 0371 Oslo Norway a.o.sjastad@kjemi.uio.no

## Abstract

Herein we report on an environmentally friendly and scalable production route for hollow silica spheres (HSSs). The process is based on close to 100% conversion of non-crystalline solid Si nanoparticles (*D̄* = 40 ± 9 nm) in mild alkaline solutions (pH ≤ 9.0) at ambient temperature. The Si nanoparticles are prepared using the centrifugal chemical vapor deposition (cCVD) method. Combining transmission electron microscopy (TEM) imaging and nanoparticle size analysis with hydrogen evolution data, elemental mapping, and nitrogen adsorption for surface area measurement, we show for the first time experimental data that document a Kirkendall type Si-to-HSS formation process. Our understanding is that the Si nanoparticles exposed to air form a SiO_2_ film, which is stable in the mild alkaline environment. Silicon from the Si nanoparticles is transported through the thin SiO_2_ film and is reacting with H_2_O/OH^−^ species on the particle surface or in the already thickened SiO_2_ shell to form silicic acid that in turn rapidly gets converted to a sol–gel to continue the growing of the silica shell. We foresee that this green chemistry approach can be utilized for HSS preparation for use in batteries, insulation materials and drug delivery.

## Abstract 2

Herein we report on an environmentally friendly and scalable production route for hollow silica spheres (HSSs).

## Graphical abstract

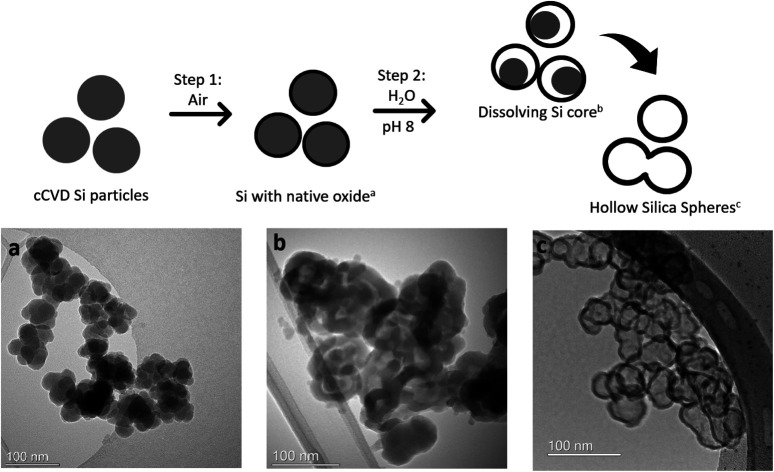

## Introduction

Hollow nanomaterials such as hollow silica spheres (HSSs) have gained significant attention due to their wide range of potential applications for thermal insulation, energy storage, catalysis, and drug delivery.^1,2^ High chemical stability, large surface area and high availability of versatile surface modification techniques make them a good candidate material for tailoring to different uses.^3^

Extensive research has led to various methods for HSS synthesis. The template method is the most widely used. Typically, this method utilizes a silica precursor that undergoes hydrolysis and condensation to form the silica shell, which in turn is deposited onto a template.^1,4^ The Stoeber process, involving the use of organic silicon compounds, is an attractive route for the purpose. After forming the shell, the template is selectively removed to create the hollow interior of the particle. Templates can range from hard materials like polymers or metal-oxide spheres to soft materials such as water-in-oil emulsions, surfactants, vesicles, polymers, or gas bubbles.^3^ Surfactants are used for mesoporous silica shell formation, utilized, for instance, for drug delivery applications. The morphology of the template material determines the shape and size of the resulting HSSs.^5^ Successful fabrication of HSSs using these methods has yielded particles within the size range of 20 nm to 10 μm.^6^ Unfortunately, harsh processing with organic solvents, high-temperature calcination, strong bases or acids are required to achieve selective etching of the different template materials.^7–10^

A few efforts have also been made to hollow out solid particles and transform silica or silicon nanoparticles into nanoshells. These methods eliminate the need for template removal but still require the use of chemical etchants to etch the inner core while preserving the outer shell selectively. Dissolution by hydrochloric acid, sodium borohydride, or other strong bases and acids, or other harsh chemical etchants has been described.^2–4,11^ For instance, crystalline Si nanoparticles made by CO_2_ laser pyrolysis of silane followed by extensive ultrasound treatment and heating the particles to 120–140 °C in water resulted in HSSs with a 4 nm shell.^12^ Others have reported HSS synthesis by use of concentrated ammonia in ethanol containing some water,^13^ or aqueous solutions with strong bases such as KOH and NaOH while observing hydrogen generation in the process.^14^

The starting material for HSS synthesis in this work is non-crystalline solid silicon (Si(0)) nanoparticles produced from monosilane (SiH_4_) *via* centrifugal chemical vapor deposition (cCVD), offering a scalable synthesis method with control over nanoparticle size and crystallinity through adjustment of synthesis parameters.^15^ Additionally, the synthesis process only gave hydrogen as the by-product, which is not an environmental threat. Advantages over conventional CVD methods include a rotational component that allows for improved synthesis control by selectively harvesting nanoparticles of a desired size range and separating them from the remaining Si produced, resulting in a narrow particle size distribution.^16^ Previous studies show that cCVD Si nanoparticles generate significant amounts of hydrogen gas in aqueous buffers with pH values ranging from 7.4 to 10.0,^15^ which can be involved in HSS formation.^14^

The aim of the present work is to investigate the suitability of non-crystalline cCVD Si nanoparticles (*D̄* = 40 ± 9 nm) as the starting material for HSS fabrication under mild pH conditions by a two-step route. Step (i) is the generation of a native oxide layer through gentle air oxidation and step (ii) is the immersion of the Si nanoparticles in slightly alkaline aqueous solutions (pH 7.4–9.0) at 37 °C. Thus, an environmentally green route is achieved with no use of organic solvents or harsh chemicals. Additionally, we propose a Kirkendall type Si-to-HSS formation mechanism with the support in experimental results that we discuss relative to relevant literature.

## Materials and methods

### Materials

Phosphate-buffered saline (PBS) at pH 7.4 was made from PBS tablets (Sigma-Aldrich). Tris buffers of pH 8.0 and pH 9.0 were made using tris(hydroxymethyl)aminomethane (Kebo Lab, VWR International) and hydrochloric acid (HCl, Riedel-de Haen, Honeywell). Ethanol and methanol were purchased from Merck.

Centrifugal chemical vapor deposition (cCVD) silicon nanoparticles were provided by Dynatec Engineering AS, and for details regarding the production see ref. 15.

### Procedure for HSS formation and H_2_ evolution measurements

The as-prepared non-oxidized cCVD Si nanoparticles were exposed to air at room temperature (RT) with a duration of minimum one week for the formation of a thin native oxide layer. Some Si nanoparticles were additionally heat treated at 300 or 500 °C (Carbolite Gero TF1-1200, Verder Scientific) in static air for 2 hours. Particles with the native oxide layer were utilized as the reference sample.

Si nanoparticles, given the various treatments in air at ambient temperature, 300 or 500 °C, were immersed in three buffer solutions (pH 7.4, 8.0 and 9.0) at a concentration of approximately 2–3 mg mL^−1^. The suspensions were kept at 37 °C under constant stirring. The volumes of H_2_ gas produced from the nanoparticles in the various buffer solutions were recorded by trapping the gas in a water-filled graduated tube. Selected samples for TEM and BET analyses were withdrawn after 50, 200 and/or 300 minutes. The withdrawn samples were centrifuged, washed once with water, or directly stored in ethanol or methanol until analysis with TEM. Samples were centrifuged and dried under vacuum overnight before BET analysis.

### Electron microscopy imaging and elemental mapping

Transmission Electron Microscopy (TEM) imaging was done by transferring the cCVD and HSS particle samples, dispersed in methanol or ethanol, onto a holey carbon-coated Cu TEM grid, and allowing them to dry in air. A vacuum transfer holder (Gatan 648) was used to load the particle samples into the TEM column. TEM images were recorded using a double spherical aberration corrected cold field emission gun (FEG) JEOL Atomic Resolution Microscope (ARM) 200FC, or using a JEOL JEM-2100F equipped with a Gatan Orius 200D CCD camera. An accelerating voltage of 200 kV was used for imaging. Both transmission and scanning imaging modes were used, giving opposite contrast.

Simultaneous analysis by Energy Dispersive X-ray Spectroscopy (EDS) and dual Electron Energy Loss Spectroscopy (EELS) was performed for elemental mapping of the samples in the Scanning Transmission Electron Microscopy (STEM) mode, using a JEOL ARM 200FC instrument. For EDS, a 100 mm^2^ Centurio detector was used, covering a solid angle of 0.98 sr. EELS data were acquired with a GIF Quantum ER imaging filter. Estimation of average elemental distribution (at%) was done from EELS data. Selected Area Electron Diffraction (SAED) was used to identify the crystalline or amorphous nature of the samples by evaluating the diffraction pattern with the electron beam interacting with the sample. STEM and EDS (with a Super-X detector) analyses were also conducted using a Thermo Fisher Scientific Cs-corrected Titan G2 60–300 kV microscope operated at 300 kV.

### Nanoparticle size analysis from TEM images

Particle size was analyzed using ImageJ software (version 1.54g). The nanoparticle sizes along the longest diameter were measured and scaled to the size of the scale bar in the same image. A total of 100 nanoparticles were measured for each sample. Particle size distribution histograms were made using Microsoft Excel (version 2207) and R studio, PBC (version 2021.09.1 Build 372), showing statistics of all measured samples (no exclusions).

### Surface area measurements

Cryogenic nitrogen adsorption isotherms were collected to quantify the surface area of cCVD Si and the HSS samples withdrawn from the H_2_-evolution experiments. A Micrometrics ASAP 2020 instrument measured the relative pressure of nitrogen as the adsorptive gas and the ASAP 2020 software calculated the surface area using the Brunauer–Emmett–Teller (BET) theory. 5–15 mg particles were degassed in BET tubes for 300 min at 80 °C under vacuum before analysis.

## Results

**Fig. 1:**
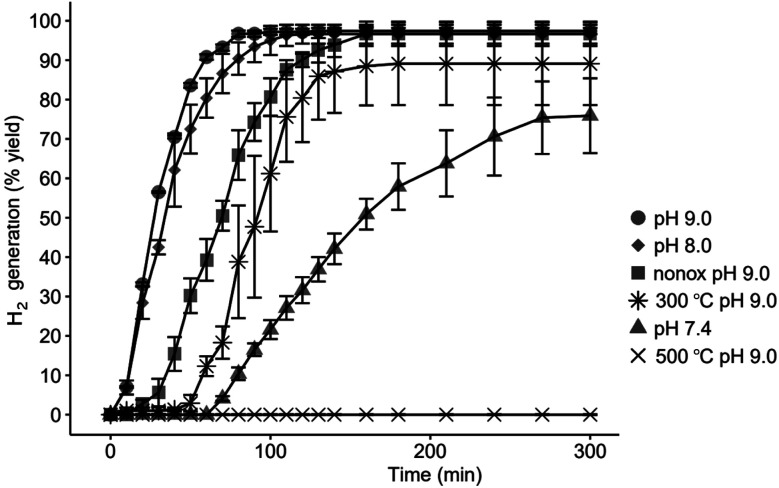
Hydrogen evolution from cCVD Si nanoparticles (*D̄* = 40 ± 9 nm) treated under different pH conditions. 100% yield corresponds to 1600 mL H_2_ from 1 g of Si (according to Si + 2H_2_O →SiO_2_ + 2H_2_). Si nanoparticles with the native oxide film in pH 9.0 (●), pH 8.0 (◆) and pH 7.4 (▲), unoxidized nanoparticles (nonox) in pH 9.0 (■) and nanoparticles heat treated in air at 300 °C (✳) and 500 °C (✕) at pH 9.0. The data are shown as the average of three parallels with error bars showing the standard deviation at each point.

The H_2_ evolution kinetics from the cCVD Si nanoparticles depends on the pre-treatment of the nanoparticles and the pH of the medium they are dispersed in, as shown in Fig. 1.

A higher pH gives nearly 100% HSS formation and a fast H_2_ evolution with a maximum of 97% yield observed for both pH 8.0 and 9.0 after 120 and 80 minutes, respectively. Pre-treatment at 300 °C in air reduces the H_2_ yield to 89% while no detectable H_2_ evolved from the sample heat treated at 500 °C. The sample not exposed to ambient air before treatment at pH 9.0 showed an unexpected, delayed response. We attribute this behavior to the hydrophobic nature of the non-oxidized Si–H terminated surface that prevents the particles from dispersing into the aqueous solution until a surface oxide is eventually formed.

**Fig. 2:**
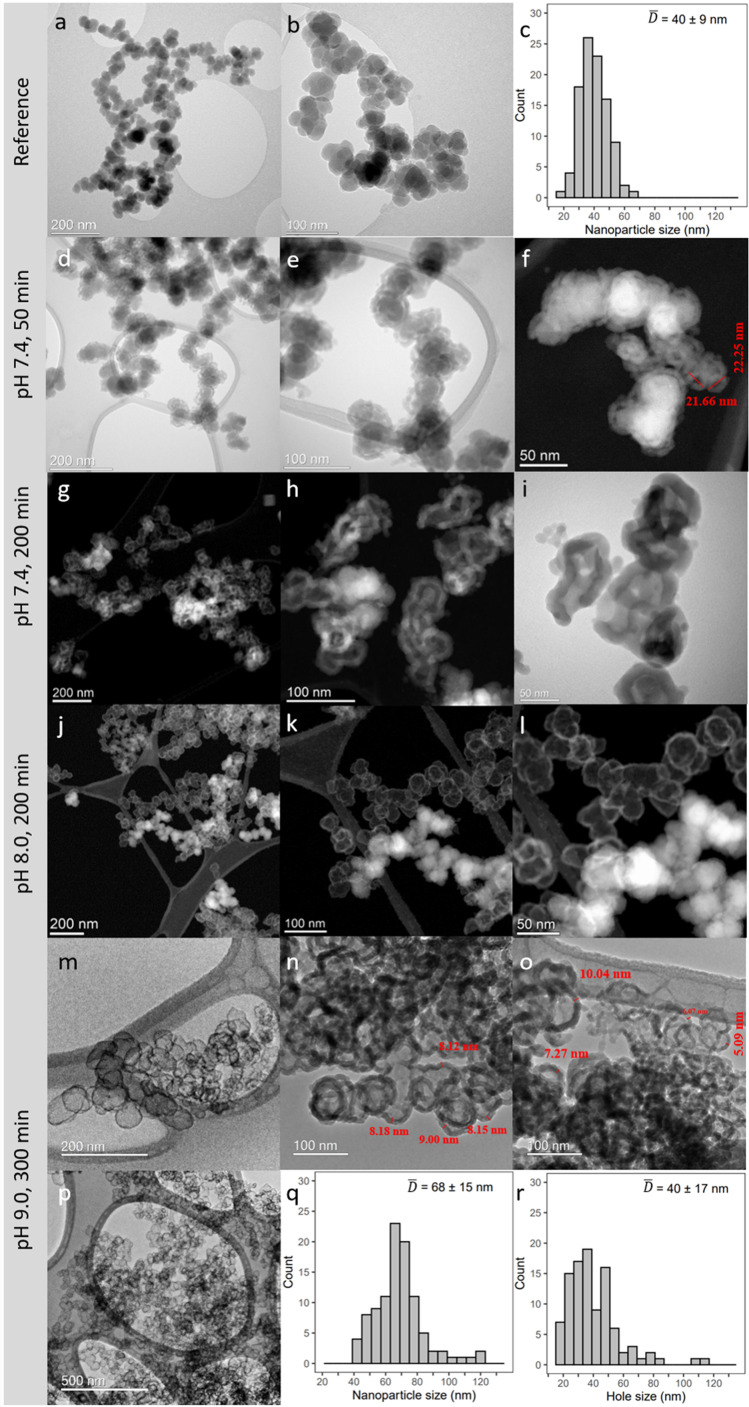
TEM images for reference cCVD Si nanoparticles (a and b) and Si nanoparticles undergoing the HSS process at pH 7.4 (d–i), 8.0 (j–l) and 9.0 (m–p) at various time intervals. For selected samples the accompanying nanoparticle size distribution histograms are included (c, q and r). Each histogram is based on analysis of 100 nanoparticles. For the pH 8.0 sample, regions are imaged to see the hollow and dense nanoparticles in one frame (j–l). At pH 8.0 and 9.0, hollow nanoparticles are the main configuration, and the TEM images showing both hollow and dense nanoparticles are not representative of the sample.

Representative TEM images with accompanying histograms from particle size analyses of the cCVD Si nanoparticles with the native oxide film (reference particles) and selected samples withdrawn from the different pH dispersions after 50, 200 and 300 minutes are reported in Fig. 2. Careful measurements of the diameters of 100 Si nanoparticles suggest that the reference cCVD Si nanoparticles have an average diameter *D̄* = 40 ± 9 nm (Fig. 2c). Note, the histogram states that some of the nanoparticles are in the size range of 10–20 nm. The Si nanoparticles are furthermore compact with no sign of hollow cores, but they posses some agglomeration or coalescence. Powder X-ray diffraction documents the reference Si nanoparticles to be amorphous (Fig. S1†).

**Fig. 3:**
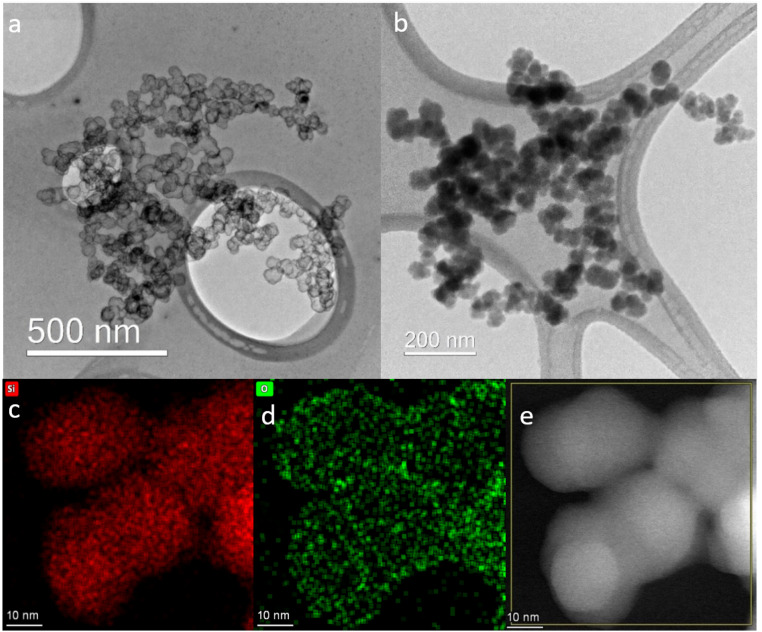
TEM images of thermally oxidized cCVD Si nanoparticles at 300 °C (a) and 500 °C (b) treated in pH 9.0. HSSs are seen for the 300 °C heat treated nanoparticles, while the 500 °C heat treated nanoparticles are solid. EDS mapping (bottom panel) of the 500 °C treated cCVD Si sample after base treatment (pH 9.0). The distribution of silicon ((c) red) and oxygen ((d) green) and the dark field TEM image of the mapped region (e).

Correlating the H_2_ evolution behavior reported in Fig. 1 with the resulting nanoparticle morphologies (Fig. 2) highlights some interesting features. At pH 7.4 and 50 min, most of the Si nanoparticles are unaffected by the mild base treatment, but a small portion has already formed HSSs with an outer diameter of ∼20–25 nm (Fig. 2d–f), in full agreement with the modest H_2_ production (Fig. 1). After 200 min exposure to pH 7.4, a 60% H_2_ yield is obtained, which aligns with observing a larger portion of HSSs and some particles showing a partial void in between the shell and core (Fig. 2g–i). After 200–300 min in pH 8.0 or 9.0 buffer solutions, a higher proportion of HSSs is observed in the TEM images (Fig. 2j–p), which explains the 97% yield in H_2_ generation. Interestingly, oxidizing the Si nanoparticles at 300 or 500 °C before base treatment affects the H_2_ production, giving H_2_ yields of 89 and 0%, respectively at pH 9.0. The TEM images shown in Fig. 3 reveal that particles post-annealed in air at 300 and 500 °C before base treatment give HSSs and solid particles, respectively. EDS elemental mapping shows that 500 °C treated nanoparticles have a homogeneous oxygen distribution throughout the particles (Fig. 3), supporting full oxidation.

**Fig. 4:**
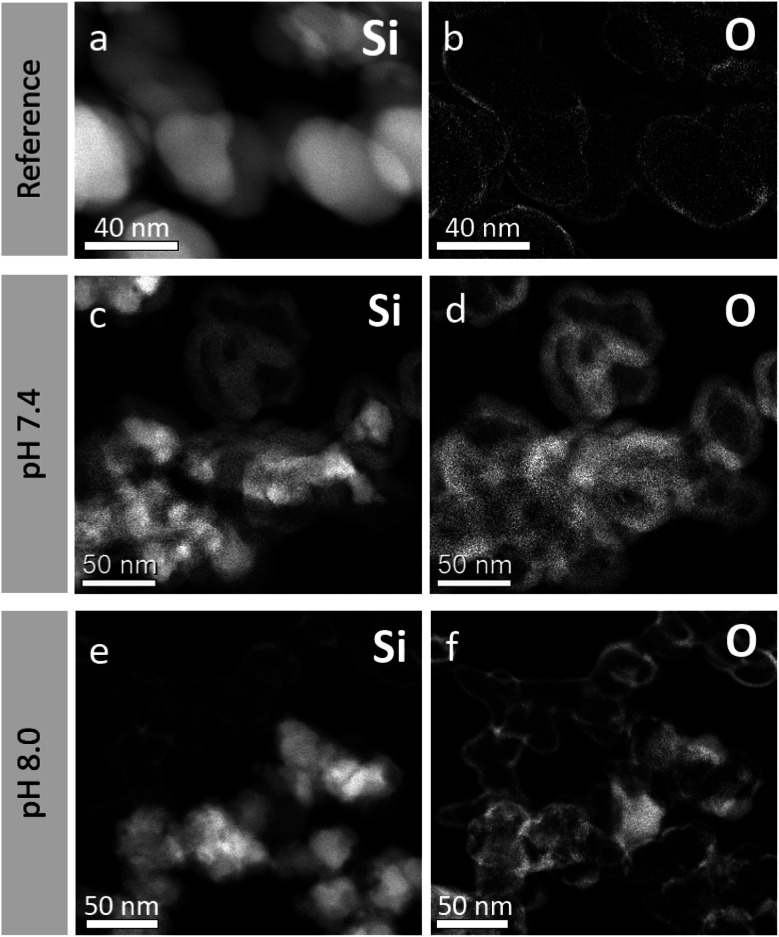
TEM images of cCVD Si reference nanoparticles (a and b), pH 7.4 treated after 200 min (c and d) and pH 8.0 treated after 200 min (e and f), with elemental analysis based on EELS data to visualize elemental distribution, Si (a, c and e), O (b, d and f). Regions are imaged to see the hollow and dense nanoparticles in one frame. At pH 8.0, hollow nanoparticles are the main configuration, and the TEM images showing both hollow and dense nanoparticles are not representative of the sample.

Returning to the cCVD Si reference nanoparticles and the HSS particles obtained from the pH 7.4 treatment for 200 min, EELS elemental analysis reveals that the shells consist of oxidized Si while the cores consist of elemental Si, see Fig. 4. The average elemental composition of the as-prepared nanoparticles reveals 6 at% oxygen and 94 at% silicon (Fig. 4a and b). This O content is attributed to the native oxide layer of 1–3 nm (see Fig. S4†) formed when exposing the as-synthesized Si nanoparticles to air at ambient temperature, giving a Si core–SiO_2_ shell like structure. The residues of the cores under basic treatment still consist of Si mainly (Fig. 4c and d). The bottom panel TEM images (Fig. 4e and f) are laid out to see both HSSs and remaining solid particles after pH 8.0 treatment for 200 min in one frame. The average elemental compositions (EELS) extracted from these particles show that the dense and hollow spheres have an oxygen content of 28 and 63 at%, respectively (the remaining is Si). Thus, the HSSs consist of Si and O in a molar ratio of approximately 1.2 : 2, close to the ratio in stoichiometric SiO_2_.

While inspecting the TEM images and histograms presented in Fig. 2 in more detail, some morphological aspects beyond the formation of HSSs are observed. Firstly, the reference Si nanoparticles with the average diameter *D̄* = 40 ± 9 nm are matching the average hole diameter *D̄* = 40 ± 17 nm of the corresponding HSSs, obtained after base treatment (exemplified at pH 9.0 for 300 min). Additionally, the corresponding obtained average HSS diameter (*D̄* = 68 ± 15 nm) is significantly larger than the diameter of the starting material. This observation, in combination with observing partly empty spheres (pH 7.4), points toward Si transportation through the native SiO_2_ film accompanied by the oxidation and thickening of the SiO_2_ shell on the outer surface or within the shell when Si is in contact with the mild alkaline solution (see Fig. 3 above). Net transport of Si out of the particles leaves behind empty voids. BET isotherms (see Fig. S5†) interestingly show that the HSS surface area is more than double that of the reference nanoparticles, on account of the formed inner and outer surface of the HSSs. The increase is seen from 56 m^2^ g^−1^ before base treatment to 127 and 162 m^2^ g^−1^ after treatment in pH 7.4 and pH 8.0 for 300 min, respectively. This shows that the nitrogen gas used in the surface area analysis can penetrate the HSSs, supporting the porosity of the HSS material. Observations of shell fragments as well as development of porosity in the formed SiO_2_ shells can also explain the increase in surface area. As a final comment on the TEM images presented in Fig. 2 for the pH 9.0 base treated sample, some HSSs are significantly larger than what is to be expected from the Si-to-HSS process itself. We suggest that these extra-large HSSs are obtained *via* fusing two or more Si nanoparticles into one larger HSS, whereof the Si nanoparticles originally were agglomerated or possessed some coalescence.

## Discussion

Herein we report an environmentally friendly and scalable production route for HSSs, based on close to 100% conversion of non-crystalline solid cCVD Si nanoparticles (*D̄* = 40 ± 9 nm) in mild alkaline solutions (pH 8.0 and 9.0) at 37 °C. To the best of our knowledge no other studies have described the formation of HSSs under such mild conditions previously. A few attempts to establish a link between the oxidation of Si in alkaline solution and the transformation into water-soluble species and subsequent formation of stable SiO_2_ like shells are previously described.^12–14^ In the following we discuss a possible formation mechanism of HSSs from solid Si nanoparticles when simply immersing them into a slightly alkaline solution and evaluate the studies described in ref. 12–14 in view of our findings.

Dissolution of elemental Si does not take place under highly acidic conditions, but at increasing pH the protective SiO_2_ film that is present on all air exposed Si surfaces becomes destabilized, and the chemical process becomes favorable with evolution of H_2_. The Si dissolution process in aqueous medium is frequently described in a simplified manner according to eqn (1).^17^

1 Si(s) + 2H_2_O(l) → SiO_2_(s) + 2H_2_(g)

For fully transformed Si nanoparticles we show that 2 mol H_2_ is produced per 1 mol Si (Fig. 1), in consensus with eqn (1). Erogbogbo *et al.* reported that H_2_ evolution depends on Si particle size (10 nm, 2.88 mol H_2_ per mol Si; 100 nm, 1.54 mol H_2_ per mol Si; Si wafer, 1.03 mol H_2_ per mol Si).^14^ Notably, other studies, *e.g*., ref. 18–20, do not reach the theoretical H_2_ yield upon reaction in mild alkaline media, implying incomplete reaction. Naturally, an incomplete reaction will give a H_2_/Si molar ratio < 2, however obtaining H_2_/Si > 2 implies release of H_2_*via* other sources. Erogbogbo *et al.* reported that the excess H_2_ release (H_2_/Si molar ratio of 2.88) from the 10 nm Si nanoparticles originates from adsorbed hydrogen on the particle surfaces from the Si production route.^14^

The fundamental criterion for shell formation is that the shell material should be more stable than the core material,^2^ which requires a difference in their chemical composition or degree of crystallinity. In our case, the as-prepared cCVD Si material has no inherent shell, but it arises once the nanoparticles form the passivated oxide layer upon exposure to ambient air. Oxidation is the key factor, and it is known that differences in the chemical composition of the silicon oxides may result in different properties.^21^ For instance, more Si–O–Si chemical bonding tends to increase the stability whereas Si–OH bonding decreases the stability.^22^ This can explain how the oxide formed in air (the shell) is more chemically resistant than the oxidized Si formed in water. This is substantiated by the finding that the oxide formed at 500 °C (likely SiO_2_) obstructed HSS formation in base, and the nanoparticles oxidized at 300 °C had a delayed H_2_-evolution response relative to Si nanoparticles with the native oxide film (Fig. 1 and 3). Note, H_2_ evolution is only possible for nanoparticles containing silicon (0) (eqn (1)), which also explains the lower H_2_ yield (pH 9.0) for the sample oxidized at 300 °C relative to the sample only having the native oxide layer.

Oxidation of Si in water, simplified in eqn (1), can be expressed through a series of equations, whereof the dominant species depend on pH, see eqn (2) and (3).

2 Si(s) + 4H_2_O(l) → H_4_SiO_4_(aq) + 2H_2_(g)

3 H_4_SiO_4_(aq) + OH^−^(aq) ⇆ H_3_SiO_4_^−^(aq) + H_2_O(l)

With increasing pH, H_3_SiO_4_^−^(aq), the reaction product in eqn (3), will continue to deprotonate in steps to H_2_SiO_4_^2−^(aq), HSiO_4_^3−^(aq) and SiO_4_^4−^(aq), implying that Si dissolution is enhanced in base, in line with our observations (Fig. 1). Notably, the reactions are also affected by factors such as salinity, reaction temperature, and Si particle/silicic acid concentration.^22^ In the present study these parameters have been kept constant except for elevated salt contents in PBS pH 7.4 compared to the other buffers (simulating isotonic biological conditions). In the pH 7.4 sample, however, complete conversion to HSSs was not observed despite the increased salt content is shifting the equilibrium described in eqn (3) to the right. Development of the shell requires deposition of a SiO_2_-like material, which occurs by polymerization of monosilicic acid (H_4_SiO_4_) into polysilicic acid (silica sol). The silica sol will be converted to a gel upon aging. The sol formation takes hours to complete at low pH whereas at pH 8–9 it is completed within seconds.^17^

**Fig. 5:**
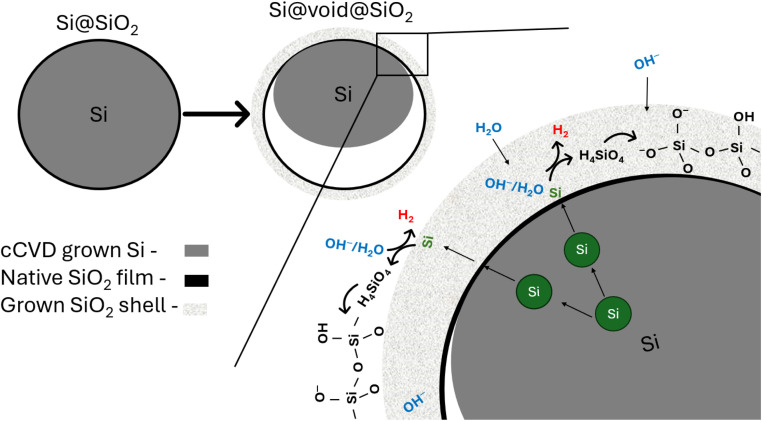
Schematic drawing of the reaction mechanism of Si-to-HSS formation. Si diffuses through the SiO_2_ native oxide layer and is oxidized by H_2_O/OH^−^ at the surface of the nanoparticles or within the oxide shell. This produces water-soluble Si species (here depicted as H_4_SiO_4_) that has increased solubility in high pH due to deprotonation (see eqn (3)), polymerize and redeposit as a SiO_2_-like structure on the outer surface or within the thickened shell. At the end of the process the core is completely etched away, resulting in hollow silica spheres (HSSs). The hollow sphere is a result of a Kirkendall type process, *i.e.*, a process where the outward diffusion (atomic Si) is faster than the inward diffusion (H_2_O/OH^−^) and the outward diffusion proceeds with vacancy formation (in the Si atomic lattice), resulting in a void/hollow sphere.

The data reported in Fig. 2 show clearly that the average diameter of the Si nanoparticles and the holes of the hollow spheres, *D̄* = 40 ± 9 nm and *D̄* = 40 ± 17 nm, respectively have a good match in size. In addition, the HSSs have *D̄* = 68 ± 15 nm. Based on these observations, we argue that silicon from the cCVD Si nanoparticles is transported from the interior of the particle through the native Si oxide layer to the surface accompanied by an oxidation process giving Si(iv) species as H_4_SiO_4_(aq). Through polymerization and gel formation the oxidized silicon grows on the outer surface of the native Si oxide film or within the formed SiO_2_ shell. The total process can be described as a Kirkendall type reaction, *e.g*., as seen when heating 150 nm solid Si particles at 950 °C in air for 5–12 hours, which gives rise to HSS formation.^23^ Bi *et al.*^23^ described the process as an initial oxidation of the Si surface to SiO_2_, giving a thick oxygen impermeable SiO_2_ layer, as a result of which Si atoms from the core diffuse through the SiO_2_ shell and become oxidized to form SiO_2_ on the surface. This is supported by an increased particle size after heat treatment. The phenomenon is well known, and observed in many systems, *e.g*., Ni is converted to a NiO shell during oxidation in air, simply because transport of Ni out of the particle is faster than oxygen transport inward to the core of the particle.^24^ Translating this to our system implies that Si atoms are transported through the native SiO_2_ film and react with H_2_O/OH^−^ species that (i) are either transported from the alkaline solution into the thickened SiO_2_ shell toward the particle core (ii) or at the surface of the particle forming SiO_2_/H_4_SiO_4_ accompanied by H_2_ evolution and subsequent gelation. Transport of atomic Si and formation of lattice vacancies is faster than transport of H_2_O/OH^−^ species in the formed SiO_2_ shell or through the native SiO_2_ film. Consequently, an empty void develops where Si was originally located. A schematic illustration of the process is shown in Fig. 5. Furthermore, if the silicic acid/sol formation had taken place on the inside of the native SiO_2_ film, no hollow spheres would be formed due to the rapid sol–gel formation (see above). Instead, a solid particle would be produced (see some additional notes to this below). Finally, the SiO_2_ shell is thickened during the process, going from a thickness of 1–3 nm (Fig. S4†) in the native SiO_2_ film to 5–10 nm (Fig. 2). This mechanism appears to be applicable to all Si nanoparticle sizes within the particle size distribution, also those <10–20 nm, since we find that Si nanoparticles exposed to pH 7.4 for 50 min show HSSs with a diameter of ∼22 nm and having holes around 10 nm (Fig. 2).

The data and the mechanism presented herein specifically concern the non-crystalline cCVD Si particles (*D̄* = 40 ± 9 nm), studied over a time range of 5 hours in mild alkaline solutions at ambient temperature. We have found three studies reporting HSS formation when exposing Si particles to an aqueous media at various pH values.^12–14^ Ref. 12 reports solely on the formation of HSSs from solid silicon particles obtained *via* pyrolysis of silane with a CO_2_ laser. However, Niu *et al.*^13^ described HSS synthesis using concentrated ammonia in ethanol containing some water (pH > 11) and commercially available crystalline Si particles of 70 nm. The proposed HSS formation mechanism includes ammonia reacting with Si, forming (NH_4_)_2_SiO_3_ as an intermediate product, which in turn deposits outside the shell by hydrolysis to grow the SiO_2_ layer. From our understanding, we would claim that the alkaline nature of ammonia promotes the dissolution of water-soluble Si species outside the particles, which becomes the driving force for dissolution of the core through transport of Si species through the SiO_2_ shell. Water is the limiting reactant to form both SiO_2_ and water-soluble Si species. Unfortunately, the study provides no insight into the size of the HSS holes *versus* Si nanoparticles, to give hints on which side of the shell the SiO_2_-like layer grows. In addition, they do not consider the rapid sol–gel formation which would promote a more solid product, if formed inside the SiO_2_ shell.^17^

Erogbogbo *et al.*^14^ applied aqueous solutions with strong bases such as KOH and NaOH when reacting batches of differently sized Si particles (10 nm; 100 nm; Si wafers) to produce H_2_.^14^ HSSs were observed as a by-product for the 100 nm Si particles after exposure to ambient air. They proposed anisotropic etching of different Si crystal planes as the mechanism for HSS formation as the shells had predominantly [111] planes parallel to their surface.^14^ This does not explain how the interior of the particles disappears, nor does it explain how the 10 nm particles simply shrunk upon dissolution without forming any HSSs. Note, in the present study, we observe that both the small (10–20 nm) and the average sized (40 nm) Si nanoparticles undergo the same dissolution mechanism, however the smaller particles appeared to be attacked first (Fig. 2f). We would argue that Erogbogbo *et al.*^14^ observed a size dependent dissolution process due to the very high pH used in their experiments and that small particles in general become more reactive/less stable and under the set conditions the small particles fully dissolved. However, repeated studies under well-described experimental conditions would be needed to explain these findings fully.

One question remains to be highlighted: how does the exchange of the water/air and/or the water-soluble Si species take place over the formed silica shell? Ref. 13, 14 and 23 claim that, if a critical SiO_2_ shell thickness is not achieved, the HSSs are permeable. HSS permeability is argued to be a requirement for their formation, allowing for free diffusion of reactants and by-products. In our studies, the BET surface area increased more than double which confirms that our HSSs are penetrable for nitrogen gas. Furthermore, in the schematic drawing (Fig. 5) of the reaction mechanism of the Si-to-HSS formation, we suggest that H_2_O/OH^−^ species are penetrating the formed SiO_2_ shell, but not the native SiO_2_ film. As stated earlier, HSS is the main configuration at pH 8.0 and 9.0. However, the schematic drawing in Fig. 5 does not account for the EELS analysis of the non-representative dense particles observed at pH 8.0 (Fig. 4), with an oxygen content of 28 at%. The increased oxygen content may be a result of more aggressive oxidation of selected Si nanoparticles when exposed to air or that H_2_O/OH^−^ species have penetrated the SiO_2_ film to partly fill the growing void and stop the HSS formation process. To get more mechanistic insight into the exact details of the dissolution of Si and the formation of HSSs and possible formation of partly oxidized dense particles, we propose *in situ* TEM and/or XPS using a liquid cell as excellent, but very challenging, approaches.

## Concluding remarks

Through this study we provide an environmentally friendly and scalable process for production of HSSs at ambient temperature and under mild alkaline conditions, starting from non-crystalline solid Si nanoparticles made by cCVD. A larger portion of HSS formation correlates with high H_2_ production, *i.e.*, at pH 8.0 and 9.0 after 120 and 80 minutes, respectively, 97% H_2_ yield and nearly 100% HSS formation. Our analyses reveal that the HSSs are amorphous and gas permeable with a wall thickness of 5–10 nm. Based on careful analysis of the TEM images of as-synthesized Si nanoparticles, HSS holes and HSS shells, we propose a Kirkendall type Si-to-HSS process. Our understanding is that the Si nanoparticles exposed to air form a thin SiO_2_ film, which is stable in the mild alkaline environment. Si is transported through the SiO_2_ film, reacting with H_2_O/OH^−^ on the particle surface or the already produced thickened SiO_2_ shell to form silicic acid which in turn rapidly gets converted to a sol–gel growing the silica shell. To the best of our knowledge, this study is the first of its kind to actively take into use experimental data to document mechanistic insight. Still, we suggest *in situ* TEM and/or XPS, with the use of a liquid cell, to get more detailed insight as a fruitful topic for further studies. We foresee that this green chemistry approach can be utilized for preparation of HSSs for use in batteries, insulation materials and drug delivery.

## Abbreviations

HSSs: Hollow silica spheres
cCVD: Centrifugal chemical vapor deposition
TEM: Transmission electron microscopy
EDS: Energy dispersive X-ray spectroscopy
EELS: Electron energy loss spectroscopy
SAED: Selected area electron diffraction
BET: Brunauer–Emmett–Teller (theory)

## Data availability

Data will be made available on request.

## Author contributions

The manuscript was written through contributions of all authors. Hennie Marie Johnsen: investigation, formal analysis, validation, writing – original draft, writing – review & editing, visualization. Anuj Pokle: investigation, formal analysis, writing – review & editing. Werner Filtvedt: conceptualization, writing – review & editing. Marianne Hiorth: methodology, writing – review & editing. Jo Klaveness: conceptualization, methodology, writing – review & editing, supervision. Anja Olafsen Sjåstad: conceptualization, methodology, writing – review & editing, supervision.

## Conflicts of interest

Werner Filtvedt and Jo Klaveness have shares in Nacamed AS.

## Supplementary Material

NA-OLF-D4NA00586D-s001
