# Supplementary material for: From silicon to silica: a green chemistry approach for hollow sphere nanoparticle formation
Source: Nanoscale Adv. 2024 Oct 11;6(24):6196–204. doi: 10.1039/d4na00586d (PMC11467762; doi:10.1039/d4na00586d)
Supplement: NA-006-D4NA00586D-s001 [file NA-006-D4NA00586D-s001.pdf]

Supplementary Information (SI) for Nanoscale Advances.
This journal is © The Royal Society of Chemistry 2024

Supporting information

From Silicon to Silica: A Green Chemistry Approach for Hollow Sphere Nanoparticle Formation

Hennie Marie Johnsen[1,2,] (h.m.johnsen@farmasi.uio.no), Anuj Pokle[3] (anuj.pokle@fys.uio.no), Werner Filtvedt[2] (wf@nacamed.com), Marianne Hiorth[1] (marianne.hiorth@farmasi.uio.no), Jo Klaveness[1] (jo.klaveness@farmasi.uio.no), Anja Olafsen Sjåstad[4] (a.o.sjastad@kjemi.uio.no)*

[1]Department of Pharmacy, University of Oslo. Sem Sælands vei 3, 0371 Oslo, Norway

[2]Nacamed AS. Oslo Science Park, Gaustadalléen 21, 0349 Oslo, Norway

[3]Department of Physics, Center for Materials Science and Nanotechnology, University of Oslo, POB 1048 Blindern, 0316 Oslo, Norway

[4]Department of Chemistry, University of Oslo. Sem Sælands vei 26, 0371 Oslo, Norway

[*]Corresponding author

Powder X-ray diffraction (XRD)

Powder X-ray diffraction analysis was carried out to determine the degree of crystallinity and possible phase content of the dry reference cCVD Si nanoparticles. A small nanoparticle sample was dispersed in ethanol and transferred to a Si zero-diffraction plate that was dried to form a thin layer before measurement. The analysis was carried out using Cu Kα radiation from a Bruker D2 Phaser diffractometer with a theta/theta goniometer and a LynxEye XE-T 1D detector. The angle (2θ) from 10 to 80° was analyzed at a step size of 0.2°. Crystalline Si powder was measured as a control sample.

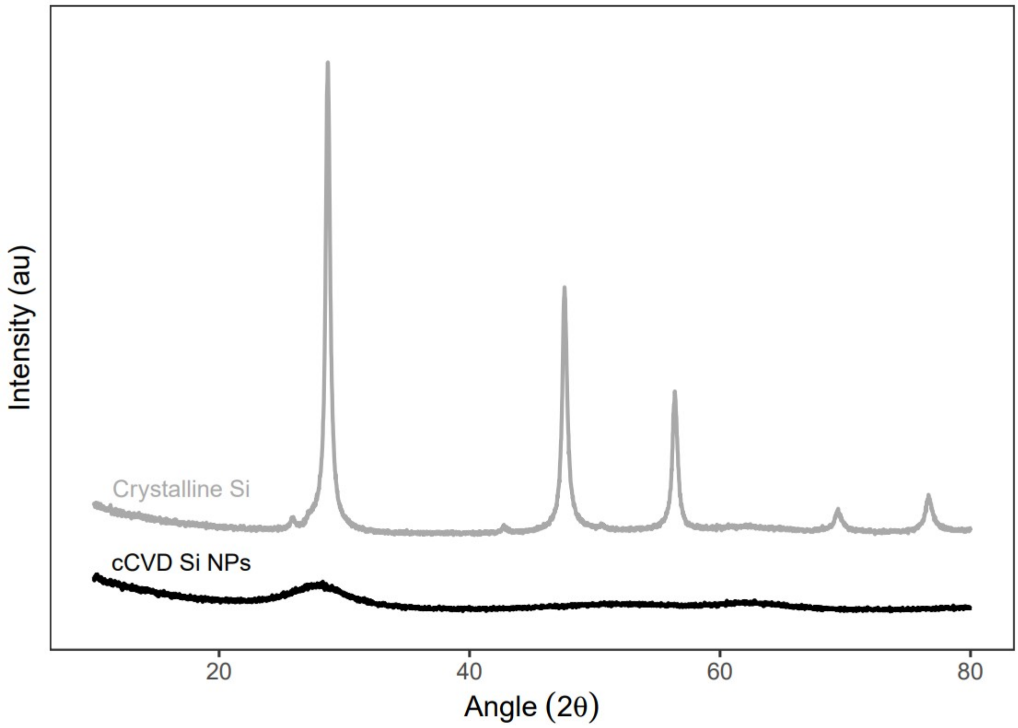

Figure S1. XRD pattern of reference cCVD Si nanoparticles shows the material to be amorphous. For comparison, the powder diffractogram of the crystalline Si sample is included.

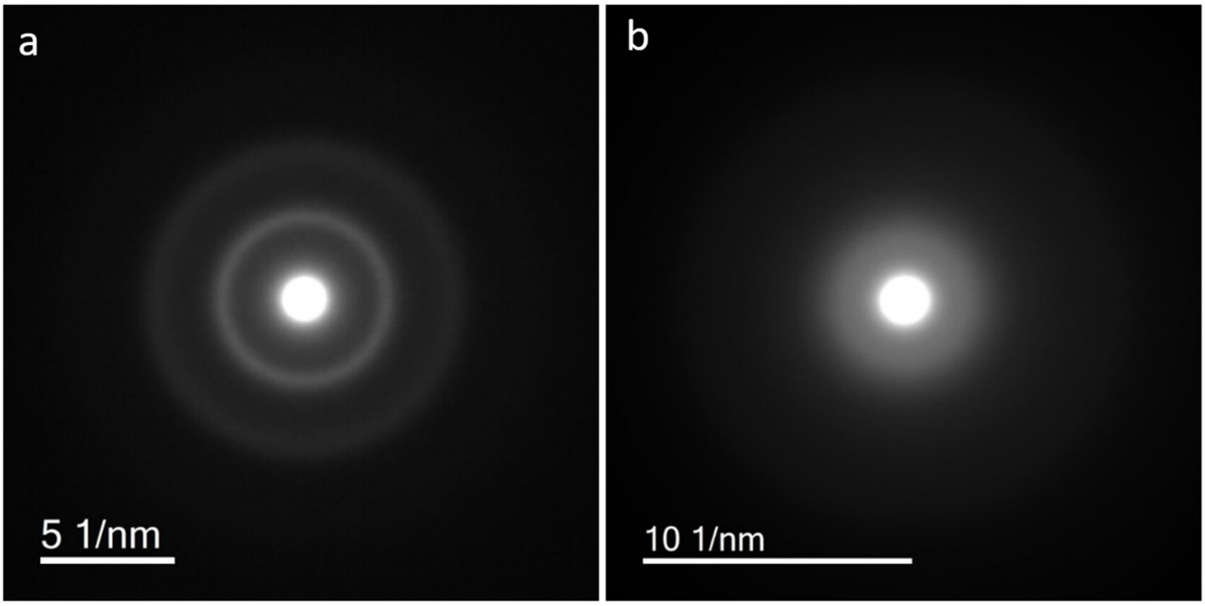

Figure S2. Selected area electron diffraction (SAED) pattern of cCVD Si nanoparticles before (a) and after (b) base treatment at pH 9.0 for 300 min, showing the materials to be amorphous.

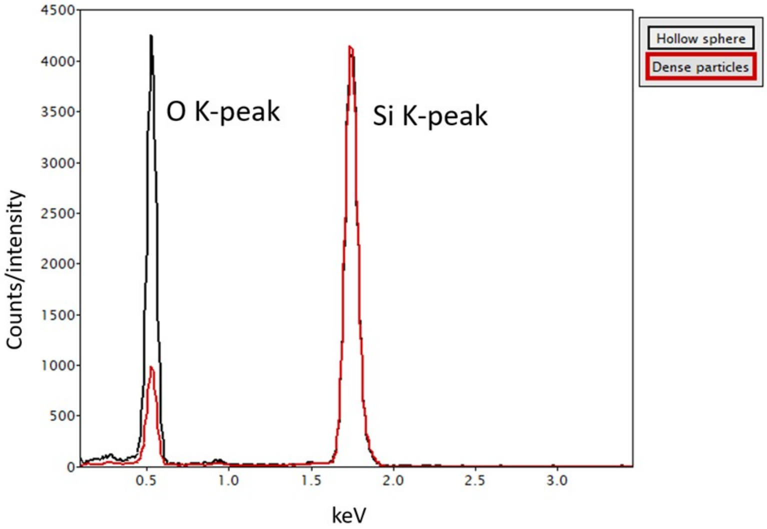

Figure S3. Electron energy loss spectroscopy (EELS) elemental analysis of dense nanoparticles and HSS seen in the pH 8.0 treated sample (Figure 4, bottom panel). Average elemental distribution from EELS analysis plotted as energy of excited photons (keV) versus counts/intensity.

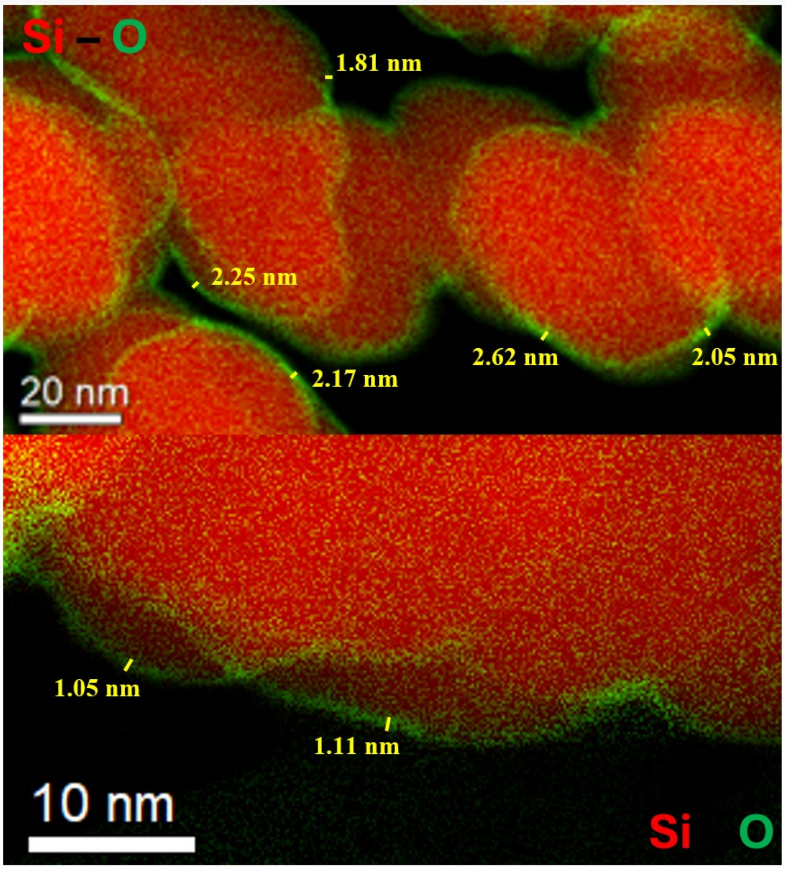

Figure S4. EELS mapping overlay of reference nanoparticles showing the distribution of Si (red) and O (green), the only elements detected in the sample. The thickness of the native oxide is indicated in several places (yellow).

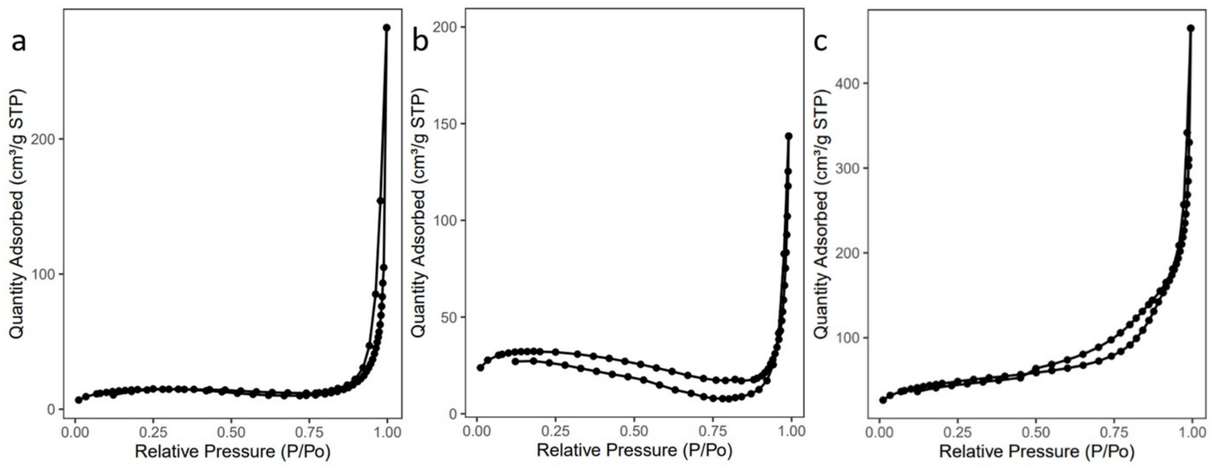

Figure S5. BET isotherms of reference Si nanoparticles before base treatment (a) and after treatment in pH 7.4 (b) and in pH 8.0 (c). These samples showed that base treatment increased the surface area from 56 (a) to 127 (b) and 162 (c) m^2/g.
